# Effects of millimeter wave irradiation and equivalent thermal heating on the activity of individual neurons in the leech ganglion

**DOI:** 10.1152/jn.00357.2014

**Published:** 2014-08-13

**Authors:** Sergii Romanenko, Peter H. Siegel, Daniel A. Wagenaar, Victor Pikov

**Affiliations:** ^1^Division of Engineering and Applied Science, California Institute of Technology, Pasadena, California;; ^2^Division of Biology and Biological Engineering, California Institute of Technology, Pasadena, California;; ^3^Neural Engineering Program, Huntington Medical Research Institutes, Pasadena, California; and; ^4^Department of Biological Sciences, University of Cincinnati, Cincinnati, Ohio

**Keywords:** millimeter wave, action potential, thermal, heating, leech ganglion

## Abstract

Many of today's radiofrequency-emitting devices in telecommunication, telemedicine, transportation safety, and security/military applications use the millimeter wave (MMW) band (30–300 GHz). To evaluate the biological safety and possible applications of this radiofrequency band for neuroscience and neurology, we have investigated the physiological effects of low-intensity 60-GHz electromagnetic irradiation on individual neurons in the leech midbody ganglia. We applied incident power densities of 1, 2, and 4 mW/cm^2^ to the whole ganglion for a period of 1 min while recording the action potential with a standard sharp electrode electrophysiology setup. For comparison, the recognized U.S. safe exposure limit is 1 mW/cm^2^ for 6 min. During the exposure to MMWs and gradual bath heating at a rate of 0.04°C/s (2.4°C/min), the ganglionic neurons exhibited similar dose-dependent hyperpolarization of the plasma membrane and decrease in the action potential amplitude. However, narrowing of the action potential half-width during MMW irradiation at 4 mW/cm^2^ was 5 times more pronounced compared with that during equivalent bath heating of 0.6°C. Even more dramatic difference in the effects of MMW irradiation and bath heating was noted in the firing rate, which was suppressed at all applied MMW power densities and increased in a dose-dependent manner during gradual bath heating. The mechanism of enhanced narrowing of action potentials and suppressed firing by MMW irradiation, compared with that by gradual bath heating, is hypothesized to involve specific coupling of MMW energy with the neuronal plasma membrane.

the use of millimeter waves (MMWs) has been rapidly increasing for a variety of over-the-airwaves applications, including high-speed wireless local area networks (Daniels et al. 2010; Verma et al. 2013), automotive driver assistance radars (Hasch et al. 2012), nondestructive testing (Ahmed et al. 2012), airport security screening (Luukanen et al. 2013), and nonlethal crowd control weapons (LeVine 2009; Woods and Ketner 2012). Since natural MMW radiation from ground-based or cosmic sources is below background ambient thermal noise power levels, or fully absorbed by the Earth's atmosphere (Salford et al. 2008), our generation is the first to become widely exposed to radiation in this wavelength band, raising questions about its interaction with the human body in general and the nervous system in particular. To address a growing public concern about the health and safety of MMW-emitting devices (World Health Organization 2010), the Institute of Electrical and Electronics Engineers (IEEE) has revised its safe human exposure standards, limiting short-term exposure to 1 mW/cm^2^ (IEEE 2005). The safety studies performed to date have relied primarily on postmortem examinations of animals that evaluated the tissue damage threshold in superficial tissue layers, including the skin (Walters et al. 2000) and cornea (Chalfin et al. 2002; Kojima et al. 2009; Kues et al. 1999; Rosenthal et al. 1976). Very few studies were performed in a living organism to evaluate the transient effects of MMWs on the nervous tissue. The existence of such transient effects on the nerve endings in fully dressed humans was demonstrated during the military testing of a nonlethal crowd control weapon that, according to a disclosed U.S. Air Force protocol, “exceeds the pain threshold but does not exceed the threshold for tissue damage” upon transient skin exposure to MMWs at 95 GHz (Gross 2010).

Our group has a long-standing interest in the mechanisms of modulatory MMW effects on neuronal excitability and activity. The effects of low MMW power density levels (<0.5 mW/cm^2^) on activity of individual neurons were first evaluated in rodent cortical slices (Pikov et al. 2010): we observed fully reversible neuronal hyperpolarization, action potential (AP) narrowing, and a bimodal effect on the firing rate, attributed to inherent heterogeneity of connectivity of sampled pyramidal neurons.

In the present study, we investigated the effect of 60-GHz low-intensity MMWs on the activity of Retzius and other identified neurons in the leech segmental ganglia (Lent 1973; Muller et al. 1981). Compared with the mammalian cortical slices, the leech midbody segmental ganglion provides a more suitable biological model for studying the mechanisms of MMW action because the intraganglionic neuronal network is considerably less complex, and therefore more deterministic (Macagno 1980). The key objectives in the present study were the identification of changes in neuronal firing and AP shape caused by MMW application, and comparison of these changes with equivalent bath heating. Our results show some unique transient effects of MMW irradiation, which can potentially be utilized for noninvasive or minimally invasive modulation of neuronal activity. Preliminary results of this study have been reported in three conference proceedings (Pikov and Siegel 2011; Romanenko et al. 2013a, 2013b).

## METHODS

Adult leeches (*Hirudo verbana*) were obtained from Niagara Medical Leeches (Westbury, NY). Groups (20–30) of animals were kept in glass aquaria with artificial pond water (36 mg/l Instant Ocean salts; Aquarium Systems, Mentor, OH) in a temperature-controlled room at 16°C and with a 12:12-h light-dark cycle. Leeches were fed with cow blood semiannually, and no feeding occurred within 1 mo of the experiments to reduce the variability in their behavior. At the time of the experiments, leeches weighed 1–3 g. Before dissection, the leech was anesthetized in ice-cold leech saline. Leech saline has the following composition (in mM): 115 NaCl, 4 KCl, 1.8 CaCl_2_, 1.5 MgCl_2_, 10 HEPES, and 10 d-glucose (all chemicals from Sigma-Aldrich, St. Louis, MO). Individual ganglia were dissected from midbody segments M6–M12 and pinned down in a Sylgard-filled dissection box. A medial dorsal longitudinal incision was made across the full body, and gut blood was flushed away. Connective tissue and the blood vessels overlaying the ganglion chain (21 total) were incised. Dissected ganglia were transferred to a petri dish and pinned down (ventral side up) in paraffin with six stainless steel pins to secure the connectives and lateral roots while leaving ∼100 μm of fluid depth under the ganglion. Before the experiment, the pinned ganglion was kept in a refrigerator (+4°C) for up to 6 h. During the experiment, the ganglion was maintained at an ambient temperature of 20–21°C, and no perfusion of fluid or oxygen was performed. For some experiments, the leech saline was modified by replacing Ca^2+^ ions with equimolar amounts of Mg^2+^ or Co^2+^ in Cl^−^-based salts. The petri dish was placed under an upright microscope (Olympus BX-FM) and filled with leech saline so that the ganglion was covered by an ∼1.5-mm-thick fluid layer. The total amount of saline was 5 ml. The ganglion was illuminated with a white LED source through the microscope optics, and a ×10 objective was used for distinguishing the neuronal types.

For electrophysiological recording from the neurons, a sharp (<1-μm diameter) intracellular electrode was fabricated from borosilicate capillary glass (0.75-mm inner diameter, 1-mm outer diameter, catalog no. 615000; A-M Systems, Sequim, WA) using a micropipette puller (P-97; Sutter Instrument, Novato, CA). The electrodes were filled with 3 M K-acetate and 20 mM KCl unbuffered solution, so their initial resistance was in the range of 22–27 MΩ. All electrophysiological recordings were performed using a microelectrode amplifier (Axoclamp 900A; Molecular Devices, Sunnyvale, CA) in the current-clamp mode, with a holding current of 0 nA, and then digitized at 20 kHz using data acquisition hardware (DigiData 1440A; Molecular Devices) and software (Clampex 10; Molecular Devices). Electrophysiological recordings were performed using the “gap-free” mode and episodic stimulation and were processed using commercial data analysis software (Clampfit 10; Molecular Devices). The firing rate and the shape of APs were evaluated for the following parameters: resting membrane potential (RMP), AP amplitude, rise and decay phases of the AP half-width (the AP span from the pre-peak half-amplitude to peak and from peak to post-peak half-amplitude), and the AP firing rate.

**Fig. 1.**
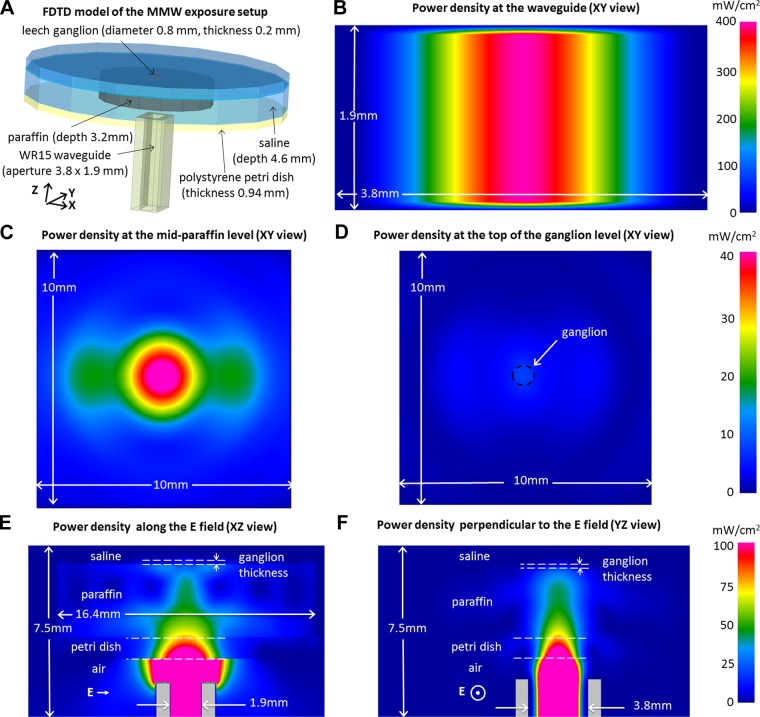
*A*: 3-dimensional finite difference time domain (FDTD) model of the millimeter wave (MMW) exposure setup. *B–D*: simulated distribution of MMW power density in several cross sections through the FDTD model taken either along (*B*, *E*, and *F*) or perpendicular to the MMW path at the paraffin level (*C*) and at the top-of-the-ganglion level (*D*). Linear pseudocolor power density scales are provided at *right* for each row of cross sections. The FDTD simulations were performed at an injected MMW power level in the waveguide of 16 mW, corresponding to an incident power density (IPD) of 4 mW/cm^2^ at the top-of-the-ganglion level.

The MMW irradiation system consisted of a synthesized microwave source for 17–23 GHz (HP 83650L; Agilent Technologies, Santa Clara, CA), a 4× frequency-multiplying stage (HP 83557A; Agilent Technologies) or an active quadrupler (AMC15; Millitech, Deerfield, MA), followed by a 20-dB amplifier (AMP-15; Millitech, Northampton, MA) to generate continuous wave power between 4 and 64 mW at 60 GHz (for the reported experiments, the powers of 4, 8, and 16 mW were used). Power levels could be controlled using internal or external attenuators and were continuously monitored with both a 60-GHz waveguide square-law detector (47344H-1200; Hughes Electronics) and a calibrated absolute thermoelectric power meter (ML83A; Anritsu, Atsugi-shi, Japan). Coupling to the ganglion was accomplished through a single-mode open-ended rectangular waveguide (WR15; 50–75 GHz band, 3.8 × 1.9-mm aperture) placed 1 mm below the petri dish bottom. The path of the MMW radiation after exiting the waveguide was through a central opening in the microscope stage, the polystyrene petri dish bottom (1 mm thick), the low-radiofrequency (RF)-loss paraffin holding the ganglion pins (3.2 mm thick), and then directly into the pinned-out ganglion (0.2 mm thick) (Fig. 1*A*). The petri dish was filled with leech saline to a level ∼1.5 mm above the top of the ganglion. The waveguide was aligned with the microscope optical axis so that the peak of the irradiating MMW beam was directed at the ganglion (the beam was much wider than the ganglion diameter; Fig. 1, *B–F*). The duration of MMW irradiation was 1 min in these experiments, and a minimal between-exposure interval of 5 min was chosen to avoid possible cumulative exposure effects.

The actual MMW power density at the ganglion level could not be directly measured, because no well-matched or calibrated MMW detector is available at a submillimeter scale for fluid-immersed measurements. Instead, the MMW power density was estimated using a finite difference time domain (FDTD) model of the experimental setup using the RF FDTD simulation software (QuickWave; QWED, Warsaw, Poland). Distribution of the MMW power density along the vertical axis and at selected horizontal cross sections were derived from the FDTD model at the irradiation frequency of 60 GHz by using the actual setup dimensions and the Agilent 85070E dielectric probe for measuring the relative permittivity (ε_r_) and conductivity (σ) values in the following materials: the polystyrene petri dish (ε_r_ = 2.56, σ = 0.003), paraffin (ε_r_ = 1.63, σ = 0.000), and leech saline solution (ε_r_ = 15.2, σ = 74.8). The ganglion was too small for us to measure its permittivity and conductivity directly, so these values were estimated to be ε_r_ = 10.9 and σ = 48.5 based on reported data for brain gray matter (Gabriel et al. 1996). A series of different mesh sizes, frequencies, geometric areas, and other parameter variations were used to confirm the absence of strong frequency-specific resonances or variations of MMW signal strength across the biological sample. Considerable power drop at and above the ganglion is accounted for by MMW beam expansion away from the waveguide aperture, reflection and refraction at each interface, and absorption in each of the transgressed materials (polystyrene, paraffin, ganglion, and leech saline). The vertical and transverse cross sections of the FDTD-estimated MMW power distribution are shown in Fig. 1, *B–F*. As a consequence of uncertainties in the physical parameters (permittivity and dimensions), the FDTD estimations are accurate only to ±6 dB. As shown in Fig. 1, *B* and *C*, the power distributions inside the rectangular waveguide and in the free space between the waveguide and saline follow the expected pattern for the fundamental mode inside and outside the guide. The power distribution is highly uniform across the small area of the ganglion as the Gaussian pattern spreads out (Fig. 1*D*). The total power loss, shown as the change from pink color at the waveguide output port level to blue color at the ganglion level in Fig. 1, *E* and *F*, corresponds to a power drop of 52×. This is due to a combination of beam spread, absorption, and interfacial reflections.

At the applied MMW powers of 4 to 16 mW, the average power density at the central area of the waveguide output port ranged from 53 to 212 mW/cm^2^ (based on FDTD estimations and consistent with the power meter measurements) and dropped to 1.0 to 4.0 mW/cm^2^ at the top of the ganglion. It should be noted that the IEEE safe exposure limit adopted by the U.S. is 1 mW/cm^2^ for a 6-min exposure (IEEE 2005).

Monitoring of the ganglion temperature during MMW irradiation was done using a fiber-optic spectrophotometric temperature-sensing device (OTG-M360 sensor with PicoM spectrophotometer; Opsens), which, unlike the typical thermal probes (Alekseev et al. 2011), is insensitive to MMW irradiation and mechanical vibration due to its optical mode of measuring the GaAs crystal bandgap. The GaAs crystal (diameter 150 μm) is affixed at the end of a 360-μm diameter fiber, allowing localized measurement of temperature at the ganglion surface with a resolution of 0.05°C and a response time <100 ms. Monitoring of the bath temperature in the bath heating experiments was done using a thermistor (TA-29; Warner Instruments, Hamden, CT) with a precision of 0.01°C while the bath solution passed through an inline heater (SH-27B; Warner Instruments) connected to a single-channel heater controller (TC-324B; Warner Instruments).

**Fig. 2.**
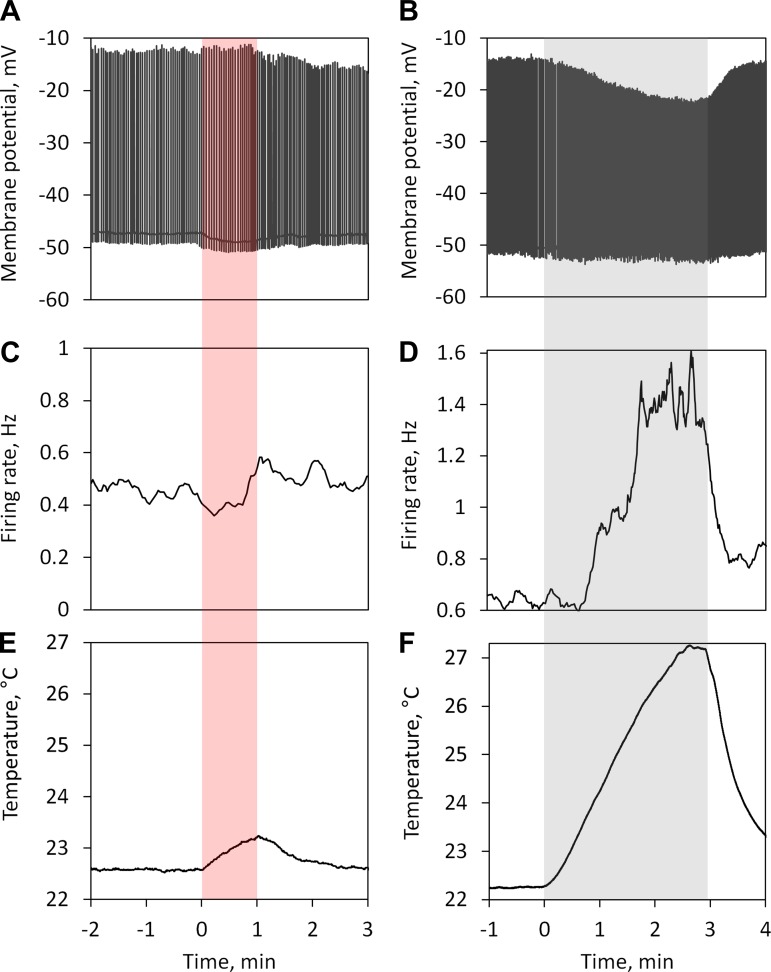
Representative recordings of the electrophysiological activity in the Retzius cell (*A* and *B*), calculated firing rate (*C* and *D*), and bath temperature (*E* and *F*) during 60-s application of MMW irradiation (60 GHz, 4 mW/cm^2^; *A*, *C*, *E*) and gradual bath heating (at a rate of 0.04°C/s; *B*, *D*, *F*). Calculation of the firing rate was based on the interspike intervals averaged for every 10 s. The shaded red area indicates the interval of MMW irradiation, and the shaded gray area indicates the interval of gradual bath heating.

The MMW effects at multiple incident power densities were analyzed using the SPSS general linear model (SPSS, Chicago, IL), followed by Dunnet one-sided post hoc *t*-tests for the postirradiation data compared with preirradiation controls. The bath heating effects were analyzed using the linear regression model (SPSS), and closeness of the fit is expressed as the adjusted coefficient of determination (*R*^2^). Comparison of the MMW and bath heating effects in Fig. 5 was performed using the one-tailed *t*-test. The significance levels of 0.05, 0.01, and 0.001 were used. The results are reported as means ± SE (except for Fig. 2, where values are means ± SD).

## RESULTS

The leech ganglion preparation was selected due to the following beneficial features: *1*) easy animal maintenance; *2*) simple dissection of the ganglia; *3*) long-term viability of the dissected ganglia due to a protective external capsule and a layer of six giant glial cells providing the ionic and osmotic balance and supply of nutrients; *4*) the large size (50–80 μm) of several ganglionic neurons, allowing easy penetration with intracellular electrodes; *5*) ready identification of large ganglionic neurons; and *6*) spontaneous firing in the ganglionic network, generated by a pair of oscillating interneurons and maintained in dynamic balance by multiple reciprocal inhibitory and excitatory loops (Cymbalyuk et al. 2002; Kristan et al. 2005). Most of the data were collected using the Retzius and “anterior pagoda” cells (61 and 27%, respectively), because these types of neurons have a large size (Mason and Leake 1978; Shan and Zhang 2001), allowing their identification in the intact ganglion under the LED pseudo-differential interference contrast light illumination and easy penetration with intracellular electrodes. Current clamp was chosen over voltage clamp because it does not suffer from space clamping-related distortions (Bar-Yehuda and Korngreen 2008). Under normal conditions, the Retzius cells have an RMP in the range of −47 ± 4 mV, AP amplitude in the range of 43 ± 10 mV, and a firing rate of about 0.6 Hz. The firing rate could be increased by intracellular current injection. However, because of the large size of the Retzius cells, inducing even a slight depolarization required a current of 0.5–1.5 nA, which might significantly alter the cell membrane properties. Therefore, all experiments were performed at a 0-nA holding current. In the leech saline, the Ca^2+^ ions were substituted with equimolar amount of Mg^2+^ ions to reduce irregular oscillatory activity, which was induced by severing the ganglion's roots and connectives during the dissection (Angstadt and Friesen 1991; Beck et al. 2002; Garcia-Perez et al. 2007).

Representative effects of 60-s-long MMW irradiation and gradual bath heating on the electrophysiological activity of the Retzius cells are shown in Fig. 2. During MMW irradiation at the highest level of incident power density (IPD) of 4 mW/cm^2^, there was a gradual hyperpolarization of the RMP and gradual decrease of the firing rate (Fig. 2, *A* and *C*), both of which returned to preirradiation levels (with some overshot) within 30 s after the MMW power was terminated. In contrast, gradual bath heating using a heater in the perfusion system produced a slow hyperpolarization and slow increase in the firing rate (Fig. 2, *B* and *D*). During the MMW irradiation at 4 mW/cm^2^, the initial heating rate (in the first 20 s) was 0.015°C and the total heating after 60 s was 0.59°C (Fig. 2*E*). In the perfusion heating control experiment, the bath heating rate was 0.04°C/s and the total temperature rise after 60 s was 2.4°C (Fig. 2*F*).

**Fig. 3.**
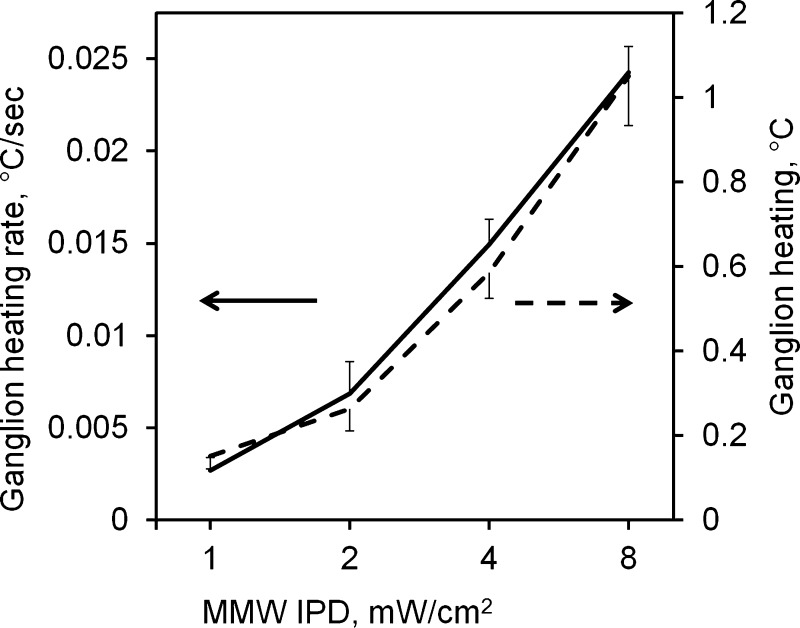
Heating rate (measured during the first 20 s) and total heating at 60 s for the leech neural ganglion during a 60-s-long MMW exposure at IPDs ranging from 1 to 8 mW/cm^2^. The temperature was measured in 22 ganglia with a fiber-optic thermal probe pressed against the top of the ganglion. Data are means ± SD.

Dynamics of the ganglion heating during MMW irradiation and bath heating were measured with a MMW-insensitive fiber-optic thermal probe (360-μm diameter) pressed against the top of the ganglion. The ganglion heating rate and the total ganglion temperature rise were linearly correlated with the log2 of the MMW IPD in the range from 2 to 8 mW/cm^2^ (Fig. 3).

**Fig. 4.**
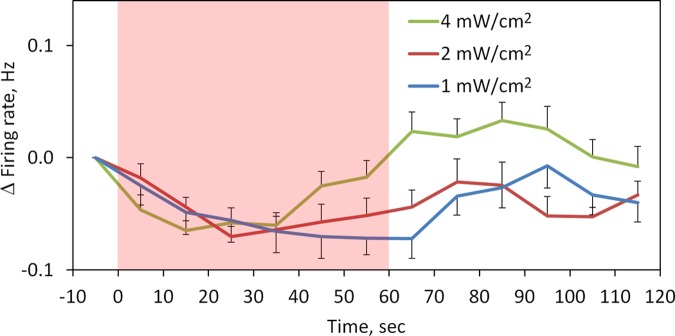
Time course of changes in the firing rate (Δfiring rate) during 60-s application of MMW irradiation at IPDs ranging from 1 to 8 mW/cm^2^. Calculation of the firing rate was based on the interspike intervals averaged for every 10 s. The shaded red area indicates the interval of MMW irradiation. Data are means ± SE.

The effect of MMW irradiation on the temporal pattern of changes in the firing rate was examined at three IPD levels (Fig. 4). At the lowest power level of 1 mW/cm^2^, the firing rate continued to decrease throughout the 60-s duration of MMW exposure, whereas at 2 and 4 mW/cm^2^, the decrease was transient and the firing rate began to return back to the baseline value at 25 to 35 s after the initiation of MMW exposure. At 4 mW/cm^2^, some increase in the firing rate was observed 50 s after the termination of MMW exposure.

**Fig. 5.**
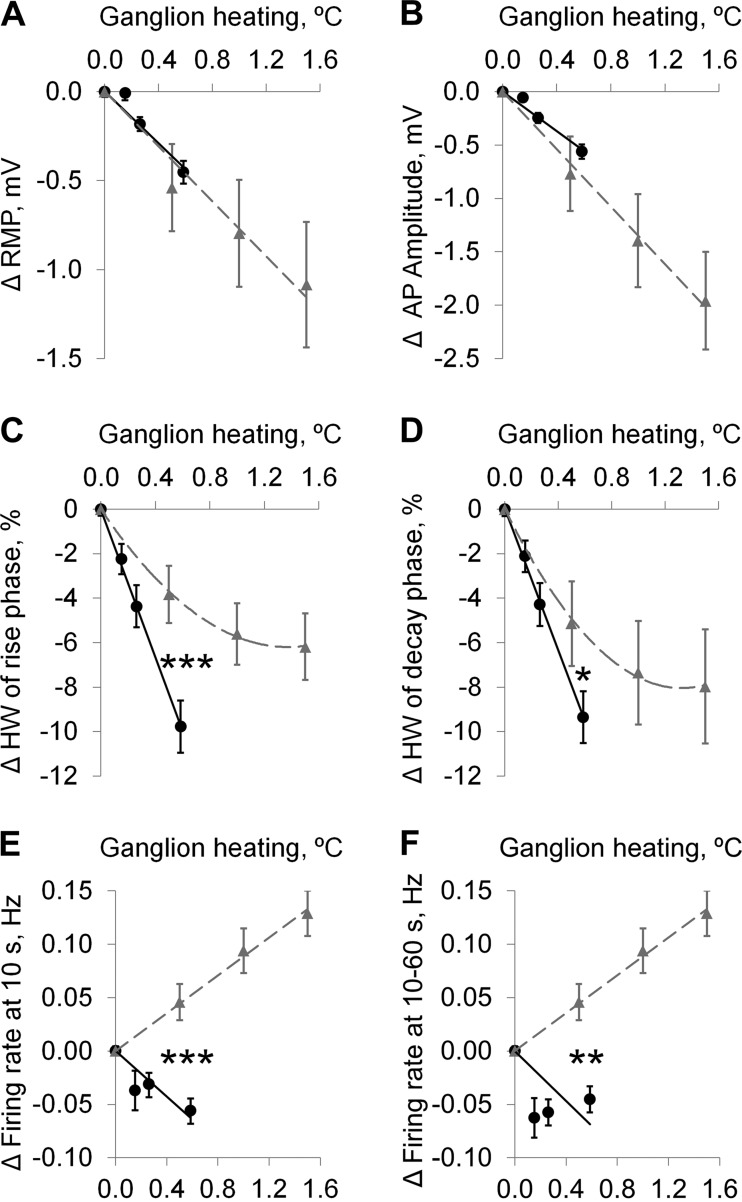
Comparison of the heating effects induced by MMW irradiation at 3 IPD levels (circles) and by gradual bath heating (triangles) on changes in the following action potential (AP) parameters in the Retzius cells: maximal change in resting membrane potential (ΔRMP; *A*), maximal change in AP amplitude (ΔAP amplitude; *B*), maximal change in half-width (ΔHW) of the rise phase (*C*), maximal ΔHW of the decay phase (*D*), Δfiring rate at 10 s after initiation of MMW exposure (*E*), and average Δfiring rate at 10–60 s after initiation of MMW exposure (*F*). Data are means ± SE. *P < 0.05; **P < 0.01; ***P < 0.001 for 1-tailed *t*-test comparing the MMW and bath heating effects at the heating level of 0.6°C. Linear regression lines are shown for MMW irradiation (solid) and gradual bath heating (dashed).

The effects of MMW irradiation (*n* = 35) and gradual bath heating (*n* = 26) on key AP parameters were compared using the calculated ganglion heating rates for MMW irradiation (Fig. 5). Ganglionic heating by MMWs and equivalent bath heating produced nearly identical dose-dependent effects on the RMP and AP amplitude (Fig. 5, *A* and *B*). In contrast, the effects of MMWs and equivalent bath heating on the narrowing of the rise and decay phases of the AP half-width were significantly different (Fig. 5, *C* and *D*). The MMW-induced effect had a linear dose dependence with 9.8 and 9.4% narrowing of the rise and decay phases, respectively, at the highest MMW IPD of 4 mW/cm^2^. The dose-dependent effect of gradual bath heating was best fitted by a logarithmic curve with 4.2 and 5.6% narrowing of the rise and decay phases, respectively, at the equivalent heating level of 0.6°C (dashed lines in Fig. 5, *C* and *D*). The changes in rise and decay times at the ganglionic heating of 0.6°C were both significantly different between the MMW irradiation and bath heating (*P* = 0.001 and *P* = 0.035, respectively). There was a MMW dose-dependent inhibition of firing rate at 10 s after initiation of MMW exposure (Fig. 5*E*); however, there was no MMW dose dependence during the remaining 10- to 60-s period of MMW exposure (Fig. 5*F*). Remarkably, the effects of the MMW irradiation and bath heating on the firing rate had the opposite polarity (Fig. 5. *E* and *F*): the MMW irradiation at all IPD levels induced a slight reduction in the firing rate, whereas gradual bath heating caused a linear dose-dependent increase in the firing rate at a rate of 0.05 Hz/°C. The firing rate changes at the ganglionic heating of 0.6°C were highly significant between the bath heating and MMW irradiation (*P* < 0.001 at 10 s and *P* < 0.01 at 10–60 s).

## DISCUSSION

This study provides a quantitative comparative evaluation of the effects of MMW irradiation and equivalent bath heating on characteristics of the APs of individual neurons. The ex vivo isolated leech ganglion preparation was selected because of its simple organization, intracellular electrode access to identifiable neurons (e.g., Retzius and anterior pagoda cells), well-defined electrophysiological and neurotransmitter properties of these neurons (Mason and Leake 1978; Shan and Zhang 2001), and a stable ionic/neurotransmitter microenvironment within the ganglionic outer capsule that is regulated by the giant glial cells (Deitmer et al. 1999). The ganglionic microenvironment was perturbed only slightly by penetrations of a micrometer-sized intracellular electrode tip (no more than 3 penetrations were performed in each ganglion to limit the ionic leakage). Intracellular recordings were made from the neurons on the ventral ganglionic side, with dorsally applied MMW irradiation passing through ∼100 μm of fluid under the ganglion and then through the ∼250-μm thickness of the ganglion. The opposed placement of the intracellular electrode and the MMW waveguide reduced direct coupling of the MMWs with the fluid inside the electrode and provided uniform MMW IPD at the ganglion.

The MMW irradiation and gradual bath heating of the ganglionic neurons produced nearly identical effects on the RMP and AP amplitude. The MMW-induced hyperpolarization was partially due to an artifact caused by the MMW heating of fluid in the glass intracellular electrode. The amount of voltage drop due to this electrode fluid heating was evaluated by inserting the electrode into a physiologically unresponsive ganglion (i.e., not exhibiting any neuronal activity). At the applied MMW IPD of 4 mW/cm^2^, the voltage drop was −0.23 ± 0.07 mV, or ∼50% of the overall hyperpolarization. The exact mechanism of this coupling is not clear but may involve MMW-induced change in the ionic gradients between the intracellular electrode and the bath solution. Regardless of the mechanism, the observed voltage-drop artifact is not relevant in quantification of the AP dynamics, which take place at a millisecond timescale, whereas the voltage-drop artifact occurred at a 1-s timescale (or even slower).

The firing rate was slightly suppressed at all applied MMW IPDs. These results are in agreement with a previous study (Alekseev et al. 1997), where the firing rate was transiently suppressed during the first minute of MMW exposure at 75 GHz and then transiently increased after the exposure was terminated. Similarly to the Alekseev et al. 1997 study, we observed an initial MMW-dose suppression of the firing rate (Fig. 5*E*), which might have been caused by an initially high thermal gradient. After 20–30 s of MMW exposure, as the heating gradient across the tissue becomes less pronounced, the exposure at higher IPD levels (2 and 4 mW/cm^2^) becomes less inhibitory. In contrast to MMWs, the firing rate was increased in a dose-dependent manner during gradual bath heating, in agreement with the results from a previous study in cockroach ganglionic neurons (Janiszewski 1986). At the equivalent heating level of 0.6°C, MMWs suppressed the firing rate, whereas bath heating increased the firing rate, a difference that was highly significant (*P* < 0.001). These results are in agreement with a previous study (Sazonov and Avelev 2000), where MMW exposure at 53 GHz and equivalent heating of 1.5°C produced opposing effects on the firing rate of electroreceptive sensory neurons (ampullae of Lorenzini) in the skate.

The MMW irradiation also produced a dose-dependent AP narrowing in the ganglion neurons, which is in agreement with our earlier study, where a similar effect was observed in the rodent cortical pyramidal neurons (Pikov et al. 2010). Interestingly, although the effect of gradual bath heating on the AP narrowing was also dose dependent, the heating of 0.6°C (equivalent of highest MMW IPD) produced only approximately one-fifth of the AP narrowing (2.1%) of that produced by the MMWs. One potential explanation for the observed differences in the effects of MMWs and gradual bath heating on the AP narrowing relates to the differences in the experimental conditions. Although our comparative experiments were performed in the same ganglion preparation, using the same bath solution, and in the same heating range, the MMW irradiation resulted in a slower overall at heating rate ranging from 0.003 to 0.015°C/s, whereas the bath heating was applied at a rate of 0.04°C/s. However, if the heating rate were, indeed, an important factor in induced AP narrowing, one would expect that a higher heating rate should result in a more pronounced AP narrowing, which was not experimentally observed. Nevertheless, the possibility that a difference in the heating rates contributed to observed differences in the AP narrowing cannot be completely ruled out.

Broadening of the AP width can be induced by incorporating lipophilic molecules in the plasma membrane (Fernandez et al. 1983; Fink et al. 2012) and is then associated with an increased DC capacitance of the plasma membrane (Fernandez et al. 1983; Wu and Santos-Sacchi 1998; Zimmermann et al. 2008). The MMW irradiation in this study produced considerable AP narrowing, suggesting a decreased capacitance of the plasma membrane. We hypothesize that the considerably stronger MMW effect on the AP width, compared with direct bath heating, is due to a specific coupling of the MMW energy with the water dipoles in the hydration layer at the neuronal plasma membrane. Analytical and numerical simulations suggest that MMWs produce a highly nonuniform electric field distribution around the neurons, with the highest field gradient concentrating at the plasma membrane (Liberti et al. 2009). The water dipoles are the key MMW absorbers in biological tissue because of their abundance and high dielectric permittivity (Beneduci 2008; Stuchly 1979). The water dipoles in the hydration layer of the plasma membrane are highly ordered (Foster and Schwan 1989; Schepps and Foster 1980; Schwan 1968; Schwan and Foster 1980), and their rotational relaxation is considerably restricted (Zhang and Berkowitz 2009; Zhao et al. 2008). We propose two (potentially interconnected) consequences of the MMW-induced disorientation of the water dipoles at the hydration layer of the neuronal plasma membrane before their rotational energy is thermalized and dissipated throughout the tissue: *1*) altered fluidity and/or structure of the transmembrane phospholipids (Beneduci 2008; Davidson et al. 2013; Ramundo-Orlando 2010) via an efficient energy transfer from the hydration layer to the membrane phospholipids (Mashaghi et al. 2012); and *2*) reduced gigahertz-range dielectric permittivity and capacitance of the plasma membrane (Blum and Henderson 1981; Grodsky 1976; Sheppard et al. 2008). Initial support for these hypotheses comes from a theoretical model describing the interaction of MMWs with biological membranes (Beneduci et al. 2014) and from experimental studies on biomembranes (Beneduci et al. 2012, 2013; Cosentino et al. 2013). We hope to evaluate these proposed mechanisms for the observed MMW effects on neuronal activity in future studies.

In conclusion, we performed a detailed comparison of the effects of MMWs and bath heating on individual neurons. We have found differences in the AP firing rates as well as in the characteristics of individual APs using these two different heating mechanisms. Whereas previous studies have monitored only the effects on compound APs from multiple neurons, we were able to quantify the parameters of individual APs. Furthermore, our study evaluated the real-time effects of the MMWs, whereas most prior studies examined only aftereffects, measured several minutes after the cessation of MMW irradiation. In the carefully controlled environment provided by the isolated leech ganglion preparation, we observed direct transient effects of the MMW irradiation on the AP dynamics, which reverted to the baseline level within 5 s after termination of MMW irradiation. The observed strong effects on the AP width could not be fully mimicked by equivalent bath heating. AP narrowing and control of firing rate by MMWs could be utilized as a noninvasive diagnostic tool or noninvasive (or minimally invasive) neuromodulation treatment modality for various neurological disorders, potentially replacing more invasive and less spatially localized electrical stimulation. Specifically, the inhibitory MMW effects can be used for pain suppression, as has been demonstrated in both animals (Radzievsky et al. 2008; Rojavin et al. 2000) and humans (Radzievsky et al. 1999; Usichenko et al. 2006).

## GRANTS

S. Romanenko was funded through a Boswell Postdoctoral Fellowship from Caltech/Huntington Medical Research Institutes. D.A. Wagenaar is funded through a Career Award at the Scientific Interface from the Burroughs Wellcome Fund.

## DISCLOSURES

No conflicts of interest, financial or otherwise, are declared by the authors.

## AUTHOR CONTRIBUTIONS

P.H.S. and V.P. conception and design of research; S.R., P.H.S., and V.P. performed experiments; S.R. and V.P. analyzed data; P.H.S., D.A.W., and V.P. interpreted results of experiments; S.R., P.H.S., and V.P. prepared figures; S.R. and V.P. drafted manuscript; P.H.S., D.A.W., and V.P. edited and revised manuscript; S.R., P.H.S., D.A.W., and V.P. approved final version of manuscript.
